# Dual Stimulus-Dependent Effect of *Oenothera paradoxa* Extract on the Respiratory Burst in Human Leukocytes: Suppressing for *Escherichia coli* and Phorbol Myristate Acetate and Stimulating for Formyl-Methionyl-Leucyl-Phenylalanine

**DOI:** 10.1155/2014/764367

**Published:** 2014-09-14

**Authors:** Izabela Burzynska-Pedziwiatr, Malgorzata Bukowiecka-Matusiak, Marzena Wojcik, Waldemar Machala, Malgorzata Bienkiewicz, Grzegorz Spolnik, Witold Danikiewicz, Lucyna Alicja Wozniak

**Affiliations:** ^1^Department of Structural Biology, Faculty of Biomedical Sciences and Postgraduate Education, Medical University of Lodz, Zeligowskiego 7/9, 90-752 Lodz, Poland; ^2^Department of Anesthesiology and Intensive Therapy, Medical University of Lodz, Zeromskiego 113, 90-710 Lodz, Poland; ^3^Department of Quality Control and Radiological Protection, Medical University of Lodz, Czechoslowacka 8/10, 92-216 Lodz, Poland; ^4^Institute of Organic Chemistry, Polish Academy of Sciences, Kasprzaka 44/52, 01-224 Warsaw, Poland

## Abstract

Although a growing body of evidence suggests that plant polyphenols can modulate human immune responses, their simultaneous action on monocyte and neutrophil oxidative burst is currently poorly understood. Based on the hypothesis that various polyphenols contained in plant extracts might affect the oxidative burst of phagocytes, we evaluated the effects of ethanolic *O. paradoxa* extract polyphenols on monocyte and neutrophil oxidative burst *in vitro* activated by different stimuli, including opsonized bacteria *E. coli*, phorbol 12-myristate 13-acetate (PMA), and formyl-methionyl-leucyl-phenylalanine (fMLP). Samples were analyzed by the dihydrorhodamine flow cytometry assay. Our results showed that the extract repressed significantly and dose-dependently reactive oxygen species production in both cell types stimulated with *E. coli* and PMA (*P* < 0.05) and its inhibitory efficiency was stimulus- and cell-type-dependent. Interestingly, there was significant stimulatory effect of the extract on bursting phagocytes induced by fMLP (*P* < 0.05). Additionally, several flavonoids and phenolic compounds as well as penta-galloyl-*β*-(D)-glucose (PGG), the representative of hydrolyzable tannins, were identified in the 60% extract by high-performance liquid chromatography (HPLC) coupled to electrospray ionization in negative ion mode. In summary, the ethanolic *O. paradoxa* extract, rich in flavonoids and phenolic compounds, exhibits dual stimulus-dependent effect on the respiratory burst in human leukocytes; hence, it might affect immune responses in humans.

## 1. Introduction

Reactive oxygen species (ROS) are highly reactive molecules including not only the oxygen radicals (i.e., hydrogen radicals HO^•^, superoxide anion radicals O_2_^•−^, etc.) but also nonradical compounds (i.e., hydrogen peroxide H_2_O_2_, singlet oxygen, etc.) that are produced in living aerobic organisms by mitochondrial electron transfer, the phagocytic NADPH oxidase, peroxisomes, and cytochrome P450 enzymes [1]. Paradoxically, ROS act as a double-edged sword in modulating human physiology. On the one hand, at low to medium concentration, they participate in the immune response, cell signaling, and apoptotic elimination of damaged cells [2]. On the other hand, their excessive production that occurs during oxidative stress, defined as an imbalance between prooxidant processes and antioxidant defense mechanisms, does oxidative damage to cellular DNA, protein, and lipids, resulting in the development of some prevalent diseases such as cancer, cardiovascular diseases, and type 2 diabetes mellitus [3, 4].

Maintaining a low concentration of ROS in cells is possible by efficiently acting antioxidant systems, including a superoxide dismutase (SOD, EC.1.15.1.1) that converts superoxide anion into hydrogen peroxide and molecular oxygen, catalase (CAT, EC 1.11.1.6) that catalyzes the conversion of hydrogen peroxide into water and molecular oxygen, and glutathione peroxidase (GPx, EC 1.11.1.19) that scavenges hydrogen peroxide in the presence of glutathione (GSH), yielding oxidized glutathione (GSSG) and water.

Over the past decade, studies on searching for dietary sources of biologically active compounds that could prevent the oxidative stress-mediated diseases received much attention. Out of them, a broad and heterogeneous group of plant polyphenols, widely available in fruit, vegetables, roots, leaves and seeds, are increasingly of interest in the field of nutrition and health due to their antioxidative, vasodilatory, anticarcinogenic, anti-inflammatory, antibacterial, and cardioprotective properties [4–6]. In this regard, the seeds of* O. biennis* and* O. paradoxa* are an important source of edible oil containing a high concentration of gamma-linolenic acid, the precursor of prostaglandin E1 and its derivatives that have been reported to affect several inflammatory diseases such as rheumatoid arthritis, eczema, inflammatory bowel disease, and multiple sclerosis, [7, 8]. Since* Oenothera* (*O. biennis *and* O. paradoxa*) defatted seeds, being a waste product of the cold-pressing production of oil, are considered as polyphenol-rich sources, a number of studies on proapoptic, anti-inflammatory, and antioxidative capabilities of their extracts have been performed in various cellular models [9–13]. In this regard, aqueous* O. paradoxa* defatted seed extract and its penta-galloyl-*β*-(D)-glucose (PGG) component have been shown to demonstrate remarkable anti-inflammatory and antioxidative properties in activated human neutrophils [11]. These activities have been attributed to their ROS scavenging abilities and their capabilities of repressing elastase, myeloperoxidase IL-8, and leukotriene B_4_ (LTB_4_) release from activated neutrophils.

Neutrophils and monocytes are phagocytic cells that represent the body's primary line of defense against invading microorganisms. In the response to various stimuli, the cells produce a large variety of ROS (O_2_^•−^, H_2_O_2_, HO^•^, hypochlorous acid (HOCl), and* N*-chloroamines (R-NHCl)) during a process called the oxidative burst in order to kill pathogens. On the other hand, excessive ROS production can be toxic to host cells causing tissue damage associated with numerous inflammatory reactions [14]. Therefore, these cells are currently believed to be important therapeutic targets in regulating inflammatory processes related to ROS generation. In this context, plant polyphenols appear to be essential modulators although their functional importance in human immune responses still remains unclear. The present study was undertaken to investigate* in vitro* the simultaneous effect of the ethanolic* O. paradoxa* extract on the oxidative burst of neutrophils and monocytes induced by various stimuli, including opsonized bacteria* E. coli*, phorbol 12-myristate 13-acetate (PMA), and formyl-methionyl-leucyl-phenylalanine (fMLP) in whole blood of healthy adults by the dihydrorhodamine flow cytometry assay. We hypothesized that the extract might regulate this process in the stimulus-activated phagocytes. In parallel, high-performance liquid chromatography-electrospray ionization mass spectrometric (HPLC-ESMS) detection was employed to identify phenolic constituents in the ethanolic extract. Additionally, the impact of the extract on the catalytic activities of major leukocyte antioxidant enzymes such as CAT, GPx, and SOD was determined.

## 2. Materials and Methods

### 2.1. Chemicals

Gallic acid, catechin, epicatechin, epicatechin gallate, ellagic acid, quercetin, caffeic acid, penta-galloyl-glucose (PGG), and methyl gallate were purchased from Sigma-Aldrich (CA, USA). Folin-Ciocalteu reagent, ethanol, EDTA, and Tris-HCl were purchased from POCH (Gliwice, POLAND). Acetonitrile, methanol, and formic acid were purchased from Bujno Chemicals (Rathburn, Walkerburn, Scotland). PHAGOBURST was purchased from GLYCOTOPE Pharma (Heidelberg, Germany). RIPA buffer was purchased from Pierce (Rockford, IL, USA). Catalase Assay Kit and Glutathione Peroxidase Cellular Activity Assay Kit were purchased from Sigma-Aldrich (USA). SOD determination kit was purchased from FLUKA (Switzerland).

### 2.2. Plant Material

Defatted and crushed seeds (100 g) of* O. paradoxa* (obtained from Agropharm, Poland) were processed by extraction with 60% ethanol (500 mL) with stirring and shaking during four hours at 51°C. Subsequently, the mixture was concentrated in a rotary evaporator (Heidolph), dried, and lyophilized.

Total polyphenol content was determined using modified Folin-Ciocalteu method. Folin-Ciocalteu reagent (10 × diluted; 2.5 mL) was added to ethanolic evening primrose extract (0.5 mL), and in the next step 7.5% sodium bicarbonate solution was added (2 mL) and all was mixed well. The entire mixture was left for 2 hours in room temperature. A gallic acid standard curve was obtained for the calculation of polyphenolic content which amounted to 55% as determined spectrophotometrically at 765 nm (LAMBDA 25 UV spectrophotometer, Perkin Elmer, UK) [15].

The* O. paradoxa* stock solution (3 mg/mL) was prepared by solving 30 mg of the extract in 10 mL of phosphate-buffered saline (PBS) containing 1% ethanol and was stored at 4°C. Under these conditions the solution was stable at least for a month, as confirmed by HPLC analysis. All experiments in this study were performed with* O. paradoxa* defatted seed extract at the final concentrations of 0.1 and 0.25 mg/mL.

### 2.3. Subjects' Recruitment

The heparinized venous blood (4.9 mL; 16 I.E. heparin/mL blood) was drawn from the healthy volunteer donors (*n* = 36; age range 25–40) at the 2nd Anesthesiology and Intensive Care Department, University Hospital No. 2 in Lodz, after the Bioethics Committee of the Medical University of Lodz approval (no. RNN/152/09/KB from 17/02/2009) and written informed consent by each subject. The including criteria were the following:

- nonsmoking;
- not taking any medication;
- absence of chronic or acute infections.

### 2.4. Leukocyte Oxidative Burst Assay

The effect of the ethanolic* O. paradoxa* defatted seed extract on monocyte and neutrophil oxidative burst was measured quantitatively by fluorometric analysis on FACS Canto II cytometer (Becton Dickinson, San Jose, CA, USA) with argon laser at a wavelength of 488 nm and with the use of a commercial kit (PHAGOBURST) in heparinized whole blood of the healthy donors. Briefly, the blood samples (100 *μ*L) were preincubated with and without the extracts (0.1 and 0.25 mg/mL) at 37°C for 30 min. Subsequently, 20 *μ*L of precooled* E. coli* suspension (unlabeled opsonized bacteria* E. coli*, 2 × 10^9^ cells/mL), 20 *μ*L of PMA working solution (1 *μ*M, final concentration), 20 *μ*L of fMLP working solution (0.6 *μ*M, final concentration), and 20 *μ*L of washing solution (a negative control) were added. After a 10 min incubation at 37°C, the fluorogenic substrate DHR 123 was added and the samples were incubated for 10 min at 37°C. To remove erythrocytes and partially fix leukocytes, 2 mL lysing solution was added and the mixtures were kept at room temperature for 20 min. After being spun down at 250 ×g for 5 min, the pellets containing leukocytes were washed with a 3 mL washing solution (PHAGOBURST) and then suspended in 200 *μ*L DNA staining solution (propidium iodide) to exclude aggregation artifacts from bacteria or cells. Cells were immediately run on the flow cytometer and were analyzed with the use of BD FACSDiva 6.0 software (Becton Dickinson). The results were expressed as the percentage of neutrophils and monocytes producing ROS (i.e., N_RFT_ [%] and M_RFT_ [%], resp.) and as a mean fluorescent intensity (MFI) which corresponds to DHR 123 oxidation per individual cell. At least 10,000 cells per sample were collected. Forward and sideways scatter (FSC and SSC) were used to select monocytes and granulocytes. To exclude any bacteria or platelet aggregates which have the same scatter light properties as leukocytes, the live gate was set in the red fluorescence histogram on those events which have at least the same DNA content as a human diploid cell. The control sample was used to set a marker for fluorescence-1 (FL1) so that less than 1% of the events were positive. The percentage of positive cells in the test samples was determined by counting the number of events above this marker position.

### 2.5. Leukocyte Protein Extract Preparation

Leukocytes were isolated from the heparinized blood (2.5 mL) of 36 healthy donors with the use of red blood cell lysis buffer as described previously [16]. The extraction of cytoplasmic, membrane, and nuclear proteins from the isolated leukocytes was performed with RIPA cell lysis buffer that consisted of 50 mM Tris-HCl, 1% NP-40, 0.25% sodium deoxycholate, 150 mM NaCl, 1 mM EDTA, 1 mM PMSF, and 1 *μ*g/mL mixed protease inhibitors such as aprotinin, leupeptin, and pepstatin (Pierce, Rockford, IL, USA). Leukocytes were suspended in 1 mL of RIPA buffer and incubated on ice for 15 minutes. After centrifuging the mixture, the rich-protein supernatant was separated and protein concentration was measured by the Bradford method [17], using bovine serum albumin as the standard.

### 2.6. Determination of Leukocyte Antioxidant Enzymes

Leukocyte protein extract (0.05 mg/mL) was preincubated with and without the* O. paradoxa* extracts (0.1 and 0.25 mg/mL) at 37°C for 30 minutes. The CAT, GPx, and SOD activities were determined with the use of commercially available kits such as Catalase Assay Kit, Glutathione Peroxidase Cellular Activity Assay Kit, and SOD determination kit, respectively.

CAT activity was measured in protein extracts at 25°C with LAMBDA 25 UV spectrophotometer (Perkin Elmer, UK) by the method described by Aebi [18]. The rate of decomposition of the H_2_O_2_ substrate was measured spectrophotometrically from changes in absorbance at 240 nm. CAT activity was expressed as micromoles of H_2_O_2_ to O_2_ per minute per milligram of protein (U/mg of protein).

GPx activity was measured indirectly by a coupled reaction with glutathione reductase (GR). Oxidized glutathione (GSSG) is produced upon reduction of H_2_O_2_ by GPx and is recycled to its reduced state by GR and NADPH (nicotinamide adenine dinucleotide phosphate, reduced). The decrease in NADPH absorbance measured at 340 nm during the oxidation of NADPH to NADP+ is indicative of GPx activity, since GPx is the rate limiting factor of the coupled reactions [19]. GPx activity was expressed as nanomoles of NADPH oxidized to NADP per minute per milligram of protein (U/mg of protein) by using the molar extinction coefficient of 6.200 at 340 nm.

SOD activity was measured by indirect method utilizing Dojindo's highly water-soluble tetrazolium salt (WST-1), 2-(4-iodophenyl)-3-(4-nitrophenyl)-5-(2,4-disulfophenyl)-2H-tetrazolium, and monosodium salt. This method uses xanthine and xanthine oxidase to generate superoxide radicals which react with WST-1 to form a water soluble formazan dye. One unit of SOD is defined as the amount of enzyme needed to exhibit 50% dismutation of the superoxide radical. All assays were performed in triplicate.

### 2.7. HPLC-MS Analysis

The qualitative analysis of ethanolic* O. paradoxa* extract was performed using Ultra-Performance Liquid Chromatograph ACQUITY UPLC I-Class (Waters Inc.) coupled with MALDISynapt G2-S HDMS (Waters Inc.) mass spectrometer equipped with an electrospray ion source and q-TOF type mass analyzer. The instrument was controlled and recorded data were processed using MassLynx V4.1 software package (Waters Inc.).

UPLC separation was done on the BEH C18 1.7 *μ*m 2.1 × 100 mm column with the gradient flow. The solvent A was 0.1% formic acid in water and the solvent B was acetonitrile. The gradient starts with 5% of solvent B to 15% in 8 min, 40% in 12 min, and 100% in 13 min and was maintained for 1 minute and then is back to the initial conditions. The UV detection was performed at 254 and 280 nm. The mass spectrometry data were acquired in negative ion mode with Capillary Voltage set to 2.5 kV.

The presence of preidentified polyphenolic compounds was confirmed using standards by comparing the retention time and high resolution mass spectra.

HPLC-MS quantitative analyses were carried out using a High-Performance Liquid Chromatograph Prominence LC-20 (Shimadzu, Kyoto, Japan) coupled with tandem mass spectrometer 4000 Q TRAP (AB SCIEX, Carlsbad, CA, USA). Mass spectrometer was equipped with an electrospray ion source (Turbo Ion Spray) and triple quadrupole/linear ion trap mass analyzer. HPLC separation was performed using a 4.6 × 150 mm XDB C8 (5 *μ*m) column (Agilent Technologies). A gradient flow was used in all measurements. The solvent A was 0.1% formic acid in water and the solvent B was acetonitrile. The gradient starts with 5% of solvent B for 3.5 minutes, then 60% in 13 min, and 100% in 14 min and was maintained for 2 minutes and then is back to the initial conditions. Multiple reaction monitoring (MRM) experiments were carried out in negative ion mode. The following precursor/product ion pairs were used: 301/65 for quercetin, 939/169 for PGG, 179/107 for caffeic acid, 301/117 for ellagic acid, 441/289 for epicatechin gallate, 289/245 for epicatechin and catechin, 183/78 for methyl gallate, and 169/125 for gallic acid. Zero air was used as the nebulizer gas and nitrogen was used as the curtain gas. The tip voltage was kept at −4.5 kV. The compound dependent declustering potential and collision energy values were set and the dwell time was 10 ms. The standard solutions of the selected polyphenols (gallic acid, quercetin, epicatechin gallate, catechin, epicatechin, ellagic acid, caffeic acid, and pentagalloylglucose) at concentrations of 0.5 ng/mL, 1 ng/mL, and 10 ng/mL (each concentration in duplicate) were prepared. The concentration of ethanolic* O. paradoxa* extract was 0.1 mg/mL and 1 mg/mL.

### 2.8. Statistical Analysis

Assessment of the variable distributions was performed with Shapiro-Wilk's test, and since they were not normally distributed, the nonparametric Friedman ANOVA test was employed. Differences between variables were calculated using Wilcoxon's test.

The results were presented as median and interquartile range (for the stimulus-activated monocyte and granulocyte oxidative burst) and as mean ± SD (for CAT, SOD, and GPx enzymatic activities).

In all analyses, the value *P* < 0.05 was considered significant. Statistical analysis was performed with a commercially available statistical software package (Statistica version 8.0, StatSoft, Poland, license no. AXAP911E504325AR-K).

## 3. Results

### 3.1. Effect of the Ethanolic* O. paradoxa* Defatted Seed Extract on Neutrophil and Monocyte Oxidative Burst

The antioxidative effect of* O. paradoxa* defatted seed extract (0.1 and 0.25 mg/mL) was investigated* in vitro* on neutrophil and monocyte oxidative burst stimulated with opsonized bacteria* E. coli* or PMA, a nonreceptor dependent activator of protein kinase C, or the weak chemotactic fMLP activator in whole blood of 36 healthy donors through the dihydrorhodamine flow cytometry based assay. This method is based on the principle that nonfluorescent dihydrorhodamine 123 (DHR 123) is oxidized to rhodamine 123, a green fluorescent compound detected by flow cytometry, through hydrogen peroxide produced during the activated cell respiratory oxidative burst [20, 21]. Therefore, the detected fluorescence is an indirect measure of neutrophil and monocyte oxidative burst.

In the current study, we employed 0.1 and 0.25 mg/mL concentrations of the extract because our preliminary flow cytometric studies performed on the blood of 10 healthy donors treated with ethanolic* O. paradoxa* extract in the concentration range from 0.1 to 1 mg/mL revealed that the extract in concentrations higher than 0.25 mg/mL caused an increase in PMA- and* E. coli*-stimulated neutrophil oxidative burst and, moreover, had no effect on monocyte oxidative burst (unpublished data). Additionally, formation of additional peaks in DNA histograms of PMA-stimulated neutrophils preexposed to the extract concentrations higher than 0.25 mg/mL was observed. Interestingly, similar but less pronounced DNA changes were also detected in PMA-treated neutrophils without the extract (control). Since the death process of neutrophils induced by PMA treatment appears to be more similar to apoptosis than necrosis in terms of an increase in the cell membrane permeability and the necessity for certain signal transductions [22], it is possible that the extract, at the concentrations higher than 0.25 mg/mL, might enhance PMA-induced neutrophil death by apoptosis.

**Figure 1:**
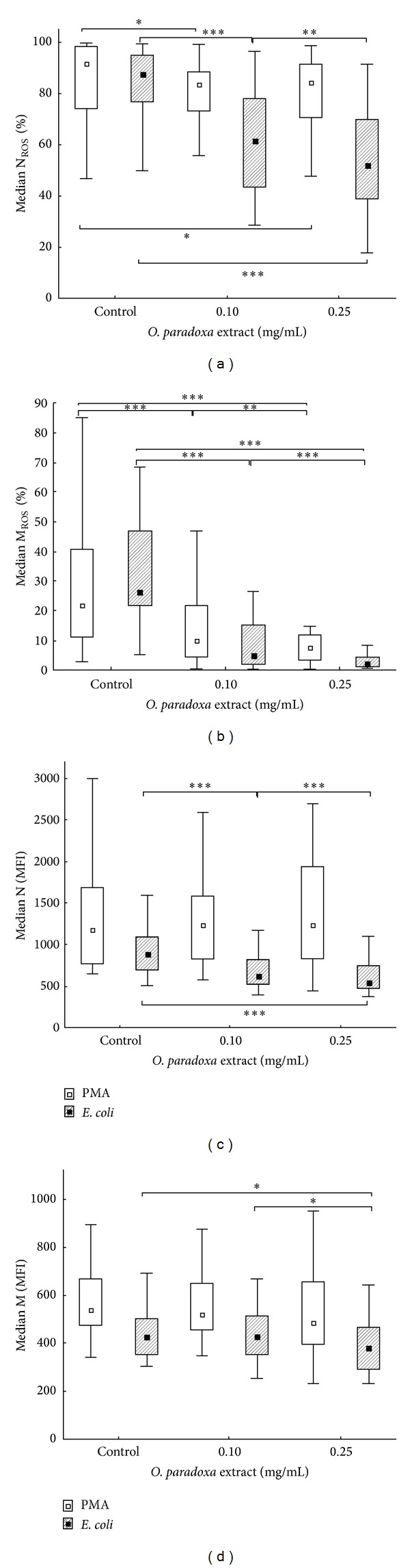
Boxplots of the percentage of neutrophils (a) and monocytes (b) producing ROS (N_ROS_ [%] and M_ROS_ [%], resp.) and a mean fluorescent intensity (MFI) of these cells ((c) and (d)) stimulated with PMA or* E. coli* after their exposition to the extract (0.1 and 0.25 mg/mL) versus control (without the extract) for PMA and* E. coli* stimuli. Middle point: median; box: interquartile range; whisker: range (excluding outliers). Significance levels *P < 0.05, **P < 0.01, and ***P < 0.001 assessed by Wilcoxon's test are indicated by asterisks.

**Figure 2:**
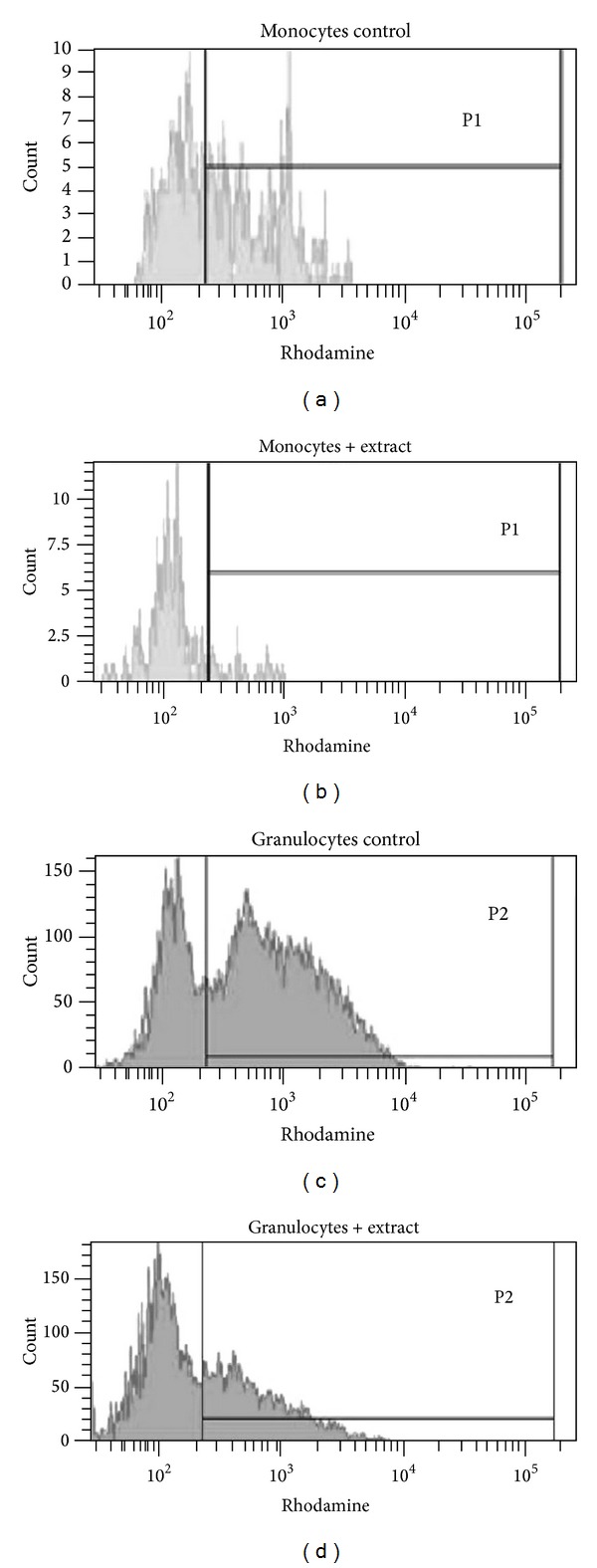
Representative histograms of the fluorescent intensities of* E. coli*-stimulated monocytes and granulocytes after* in vitro* exposure to 0.25 mg/mL extract versus the controls (without the extract). P1-monocytes, P2-granulocytes.

Table 1 is a summary of the results obtained from the flow cytometry assay. There was a stimulus-dependent effect of ethanolic* O. paradoxa* defatted seed extract on phagocytic cell oxidative burst, reflected by differences in the median of ROS-producing monocytes and neutrophils (M_ROS_ [%] and N_ROS_ [%]) activated by opsonized* E. coli*, PMA, or fMLP after their exposure to the extract at 0.1 and 0.25 mg/mL concentrations as well as of the MFI values of these cells. The extract repressed significantly both* E. coli*- and PMA-induced oxidative burst in monocytes and neutrophils (*P* < 0.05). As it was seen in* E. coli*-activated monocytes pretreated with the extract, the median fraction of ROS producing cells was markedly decreased, dependently on the extract concentration when compared to control (*P* < 0.001) and the M_ROS_ [%] values were reduced 5.2- and 12.4-fold at 0.1 and 0.25 mg/mL concentrations of the extract, respectively (Figure 1(b)). The median MFI of* E. coli*-activated monocytes exposed to 0.25 mg/mL concentration of the extract was also significantly lower compared to control (361* versus* 426; *P* = 0.023) (Figure 2).

For* E. coli*-stimulated neutrophils, the extract decreased significantly and dose-dependently the median fraction of ROS-producing cells (*P* < 0.01), but its inhibitory effect was weaker than that observed in monocytes (Figure 2). Consistent with this, the N_ROS_ [%] values were reduced 1.4- and 1.7-fold at 0.1 and 0.25 mg/mL concentrations of the extract, respectively, compared to control. The median MFI of* E. coli*-activated neutrophils pretreated with the extract was decreased significantly and concentration-dependently as compared to control (619 and 550 for 0.1 and 0.25 mg/mL of the extract, respectively,* versus* 894; *P* < 0.001).

Thus, the extract markedly repressed* E. coli*-activated monocyte and neutrophil oxidative burst, but its inhibitory effectiveness on ROS-producing phagocytic cells was, surprisingly, more pronounced in monocytes than neutrophils despite the fact that neutrophil oxidative burst in response to this activator was generally, as expected, stronger than in monocytes.

The antioxidative activity of the extract on PMA-activated oxidative burst in the phagocytic cells was lower than that seen with* E. coli* stimulus. The median fraction of ROS-producing monocytes preexposed to the extract was significantly decreased in a concentration-dependent manner compared to control, and the M_ROS_ [%] values were reduced 2.2- and 2.8-fold at 0.1 and 0.25 mg/mL concentrations of the extract, respectively. In the case of activated neutrophils, a decrease in the median of ROS-producing cells was 1.1-fold compared to control, irrespective of the extract concentration. No statistically significant changes were detected in the median MFI for both leukocyte cell subpopulations (*P* > 0.05).

In contrast to the inhibitory properties of the extract on* E. coli*- and PMA-induced monocyte and neutrophil oxidative burst, the extract was able to stimulate this process in the phagocytic cells activated by fMLP. The median fraction of ROS-producing monocytes and neutrophils after fMLP stimulation was very low (0.3 and 3% for monocytes and neutrophils, resp.) and its slight, but statistically significant, increase was seen when the cells were preexposed to the extract. Thus, ethanolic* O. paradoxa* defatted seed extract has a dual action, depending on the stimulus. On the one hand, it can suppress the oxidative burst in phagocytic cells induced by strong activators such as opsonized* E. coli* and PMA, whereas, on the other hand, the extract can stimulate it when a weak stimulus such as fMLP is used.

### 3.2. The Impact of the Extract on Leukocyte Antioxidant Enzyme Activity

To determine whether the ethanolic* O. paradoxa* extract affects leukocyte CAT, GPx, SOD activities, it was preincubated (at 0.1 and 0.25 mg/mL final concentrations) at 37°C for 30 minutes with leukocyte protein extracts prepared from heparinized whole blood of 36 healthy adults and, in turn, the antioxidant enzyme activities were determined according to the procedures described in Materials and Methods. As shown in Table 2, no significant differences in the catalytic activities of leukocyte CAT, GPx, and SOD were found between the extract-treated samples and controls without the extract (*P* > 0.05).

### 3.3. HPLC-MS Analyses of the Extract Polyphenolics

Large variety of polyphenolic compounds were found in the extract from the defatted seeds of* Oenothera paradoxa* such as phenolic acid derivatives, flavonoids, procyanidins (B1, B2, and B3), and gallotannins. The presence of the gallic acid, catechin, epicatechin, epicatechin gallate, ellagic acid, quercetin, caffeic acid, penta-galloyl-glucose (PGG), and methyl gallate was confirmed and they were quantified using standards. Initially used gradient HPLC method taken from literature was modified due to the UHPLC-MS requirements and for the fast quantitative analysis.

**Figure 3:**
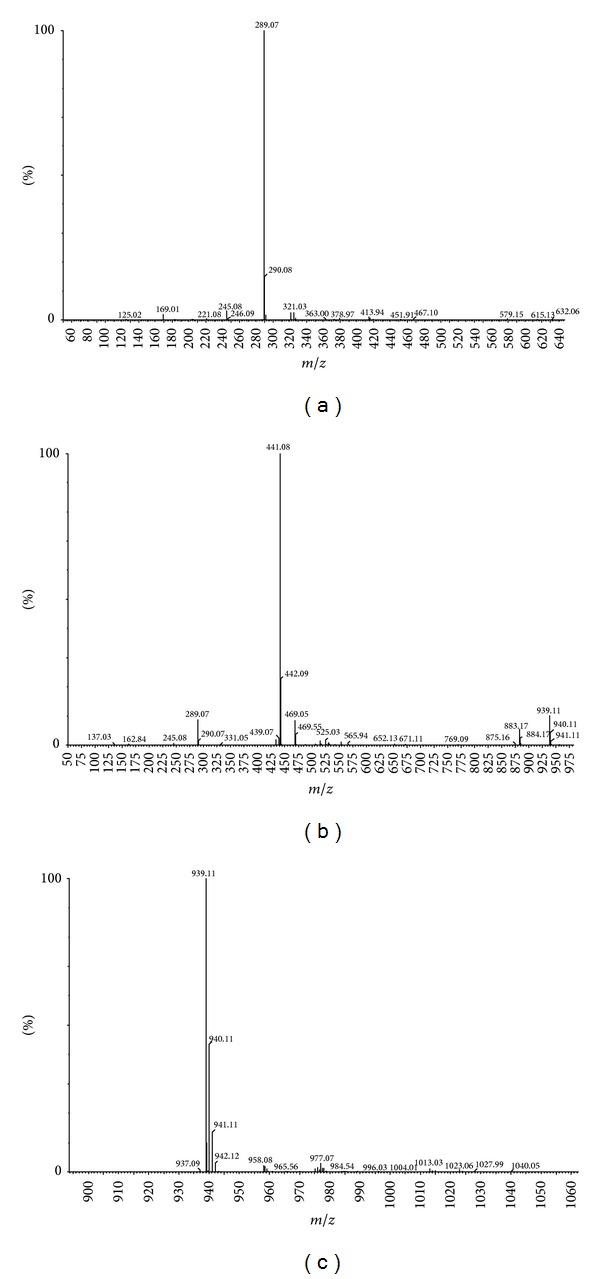
Mass spectra for catechin, *m*/*z* 289.07 (a), epicatechin gallate, *m*/*z* 441.08 (b), and PGG, *m*/*z* 939.11 (c) obtained with negative ion electrospray ionisation.

The main peaks in the chromatogram are related to gallic acid present at 1.62 min (the ESI-MS spectrum shows the deprotonated ion (*m*/*z* 169) and fragmentation ion (*m*/*z* 125) that originate in the decarboxylation process); catechin present at 4.77 min with the deprotonated ion (*m*/*z* 289); Figure 3(a) present in the spectrum (further distinguished from traces of epicatechin using standards); epicatechin gallate (10.25 min; Figure 3(b)) and isomers (8.22 and 9.92) with the deprotonated ions *m*/*z* 441 and the same molecular formula, according to the high resolution measurement; penta-galloyl-glucose present at 10.32 min with the deprotonated ion (*m*/*z* 939) present in the spectrum (Figure 3(c)). The other compounds including quercetin (12.04 min, *m*/*z* 301), ellagic acid (9.55 min, *m*/*z* 301), methyl gallate (4.53 min, *m*/*z* 183), and caffeic acid (5.54 min, *m*/*z* 179) were also found in the ethanolic* O. paradoxa* extract and their molecular formulas were confirmed by the high resolution measurements. All the above-mentioned compounds were confirmed using standards and quantified and the data were summarized in Table 3.

On the basis of the peak area, the percentage of each of the confirmed polyphenols in the dry mass of ethanolic* O. paradoxa* extract was calculated. Catechins are the most abundant polyphenols (4.8%), whereas PGG claimed in the literature [11, 23] as the most active polyphenol in this extract is only 1.66% of the dry weight.

## 4. Discussion

Over the last years, natural compounds of plan origin have been extensively studied* in vitro* and* in vivo* and many of them are currently considered as attractive candidates in therapeutic development. In this regard,* O. paradoxa* defatted seed extract polyphenols have been demonstrated to have beneficial antitumor, anti-inflammatory, and antioxidative properties [9, 11, 24]. Although antioxidant capacity of* O. paradoxa* defatted seed extract has been demonstrated to attenuate human neutrophil oxidative burst* in vitro* [11, 24, 25], its ability to reduce simultaneously the oxidative burst in human neutrophils and monocytes has not been elucidated in the literature. To address this gap, we conducted the present study with the use of the whole blood model which appears to be ideal to evaluate antioxidant capacity of the extract since isolation of the leukocyte subpopulations from the blood requires time-consuming methods and may alter their normal function [26]. We also employed the dihydrorhodamine flow cytometry based assay which enables the analysis of heterogeneous cell populations individually and simultaneously in a small amount of whole blood through differences in their light scattering properties. This method is based on the conversion of nonfluorescent dihydrorhodamine 123 substrate into fluorescent rhodamine 123 product by intracellularly generated H_2_O_2_ [20, 21].

The present study has revealed several interesting and novel findings concerning modulating properties of the ethanolic* O. paradoxa* defatted seed extract on the oxidative burst in various stimulus-induced monocytes and neutrophils. Of particular importance is that the extract exhibited a dual action on activated phagocytic cells, depending on the stimulus. When a strong activator such as opsonized bacteria or PMA was used, the extract markedly suppressed the respiratory burst in both monocytes and neutrophils as indicated by the significantly elevated level of ROS-producing cells as well as their MFI values (*P* < 0.001, Table 1 and Figure 1). On the other hand, the extract did not reduce but significantly increased the percentage of ROS-generating phagocytes after their activation by the weak fMLP stimulus (*P* < 0.05, Table 1). Regarding inhibitory capability of the extract, its more pronounced effect was seen in opsonized* E. coli*-stimulated phagocytic cells than in PMA-stimulated phagocytic cells (Table 1; Figures 1 and 2). This disparity may stem from the interference of the extract polyphenols with diverse stimulus-activated intracellular signaling events resulting in ROS production rather than their direct ROS scavenging activity. Accumulating evidence indicates that different stimulatory agents can act via multistep signaling mechanisms leading to NADPH oxidase activation, a key enzyme engaged in the oxidative burst of phagocytes that catalyzes the reduction of oxygen to superoxide anion [27, 28].

The enzyme consists of the membrane-bound flavocytochrome* b*_558_ with gp91phox and p22phox subunits and the cytosolic water-soluble proteins such as p47phox, p67phox, p40phox, and Rac1 [29]. When the cells are exposed to appropriate stimuli, the cytosolic components migrate to the membrane, where they assemble with the flavocytochrome* b*_558_ to form the active enzyme. The precise mechanisms of stimulus-mediated NADPH oxidase activation are complex and are not yet completely understood. In this regard, the opsonized particles, recognized by complement and immunoglobulin receptors localized on the cellular surface of leukocytes, have been shown to initiate phagocytosis and trigger NADPH oxidase activation through phosphorylation of its cytosolic components p47phox, p67phox, and p40phox catalyzed by numerous protein kinases, including protein kinase C (PKC) isoforms, mitogen-activated protein kinases (MAPKs), cAMP-dependent protein kinases, and protein tyrosine kinases [27]. Unlike opsonized bacteria, the phorbol ester PMA is a nonreceptor stimulus of the oxidative burst which has been demonstrated to activate the NADPH-oxidase through redistribution of PKC and phosphorylation of its cytosolic component p47phox [28]. Given NADPH oxidase inhibition by polyphenols has been proposed as an important mechanism of their antioxidant effect in stimulus-induced neutrophils [30, 31], differences in inhibitory activity of* O. paradoxa* extract between opsonised* E. coli*- and PMA-induced monocyte and neutrophil oxidative burst found in the current study could point to differences in stimulus-induced intracellular signaling responsible for NADPH oxidase activation. Therefore, it will be necessary to elucidate in further studies the signaling mechanisms underlying inhibitory activity of the ethanolic* O. paradoxa* extract on phagocytes in response to their* E. coli* and PMA activation which will help us to understand the role of the extract polyphenols in this process.

It is now well known that activated neutrophils generate both extra- and intracellular ROS [32].

In the present study,* O. paradoxa* extract attenuated the oxidative burst in opsonized* E. coli*- and PMA-stimulated monocytes more than neutrophils although its level was generally seen higher in neutrophils than monocytes activated by these stimuli. It should be noted that, surprisingly, the extract utilized in the present study attenuated the oxidative burst in opsonized* E. coli*- and PMA-stimulated monocytes more than neutrophils although its level was generally seen higher in neutrophils than monocytes activated by these stimuli. Despite the fact that the reasons for this are currently unknown and no definite conclusions can be drawn until more studies in this field have been performed, it is likely that the oxidative burst in monocytes rather than neutrophils induced by whole bacteria in the circulation (*in vivo*) would be more sensitive to the extract activity.

In contrast to suppressing properties of the extract [11], its stimulatory effect on the bursting phagocytes induced by fMLP was observed in the present study. The fMLP is a well-known leukocyte chemoattractant which binds to formyl peptide receptors coupled to guanine nucleotide regulatory proteins (G proteins) and activates several important signal transduction pathways that, in turn, cause biochemical responses accountable for physiological defence against bacterial infection and cell damage, including phospholipases C, D, and A_2_ (PLC, PLD, and PLA_2_), phosphatidylinositol-3-kinase (PI3K), PKC, and MAPKs [33]. In line with this, it may be assumed that stimulating properties of the extract might stem from its direct influence on fMLP binding to the receptors, altering some of the signaling events that promote NADPH oxidase assemblage [34]. This mechanism might be of potential relevance to the expression of biological functions of monocyte and neutrophils where fMLP-mediated reactions are involved. However, the sequence of events by which the extract might affect stimulation phagocytes activated by fMLP requires further detailed studies.

It is noteworthy that fMLP, at 0.6 *μ*M concentration, generated very low level of the oxidative burst as measured by flow cytometric assay in the present study (Table 1). This is consistent with the results found by O'Gorman and Corrochano [35], in which fMLP used at the concentrations from 10^-6 ^M to 10^-4 ^M caused very low fluorescence of granulocytes in whole blood model. Our results do not corroborate those obtained by Kiss et al. [11] who reported strong inhibitory effect of* O. paradoxa* extract on fMLP-induced ROS production in human neutrophils. The discrepancy may be explained by different experimental cellular models and the extract used to assess the oxidative burst by Kiss et al. [11] who employed isolated neutrophils and the aqueous extract as opposed to whole blood and the ethanolic extract used in the present study. Research conducted by Koffi et al. [36] suggests that ethanol is the most efficient solvent for the extraction of plant polyphenolic components.

Antioxidant enzymes such as CAT, GPx, and SOD are the principal components of the antioxidant system engaged in the maintenance of an optimal redox balance and their deficiencies can lead to oxidative stress and tissue damage. Mounting evidence supports a substantial role of polyphenols to prevent or attenuate decreases in activities of these enzymes in animal models of oxidative stress [37]. Interestingly, in this study we found that the ethanolic extract did influence leukocyte CAT, GPx, and SOD activities under normal circumstances, that is, when ROS production was not elevated. These results are in line with previous studies where no effect of wine polyphenols was observed on the catalytic activities of these enzymes in the liver, kidney, and lung of Wistar rats (rats without pathologies) [38]. However, since antioxidant effects of polyphenolics seem to be related to the oxidative status of the organism, we cannot exclude the possibility that the extract might affect the catalytic activities of these enzymes in conditions of elevated ROS production during the oxidative burst of phagocytes.

Plant polyphenols comprise a wide variety of compounds that are based on the number of phenol rings contained there and the structural elements binding these rings categorized into four main classes, including phenolic acid, flavonoids (divided into six subclasses such as flavones, flavonols, flavanones, flavanols, anthocyanidins, and isoflavonoids), stilbenes, and lignans [39]. The phenolic composition of both* O. biennis* [13] and* O. paradoxa* [23] seed extracts has been investigated previously and the presence of several flavonoids (i.e., catechin, epicatechin, epicatechin gallate, procyanidins, and quercetin) and phenolic compounds (i.e., gallic, ellagic, and caffeic acids) and PGG, the representative of hydrolyzable tannins, was detected [23]. The high-resolution HPLC-MS analysis of the 60% ethanolic* O. paradoxa* extract performed in the present study confirmed the composition of polyphenol components contained in the extract, except procyanidins (Table 3). Unfortunately, due to having no available standards, we could not confirm the existence of this flavanol with different degree of polymerization in the extract. However, taking into account the fact that the total polyphenol content in the ethanolic* O. paradoxa* extract was 55% and the identified polyphenols comprised 15% (Table 3), we cannot rule out the possibility that 30% of the remaining extract polyphenolics would be procyanidins, particularly since the HPLC-UV analysis of the extract constituents in our study showed the presence of a wide peak characteristic for polymeric proanthocyanidins that reflected their high content (data not shown) [23]. It is noteworthy that catechin was the major component among the identified polyphenols in the* O. paradoxa* extract, according to previous studies [9, 11, 23, 40]. Catechins are well-known bioactive compounds and potent antioxidants as has been described in* in vitro* and* in vivo* studies [4, 41]. However, in the current work, we did not use the pure catechin to evaluate its antioxidative power towards the oxidative burst of* E. coli*- and PMA-stimulated phagocytes since the total activity of the extract can be affected by synergism and/or antagonism between phenolic compounds; thus, there was a need to prove that these beneficial effects are derived from the polyphenols contained in the extract.

## 5. Conclusion

In our study we can conclude that inhibitory efficiency of the ethanolic* O. paradoxa* extract towards ROS production in activated phagocytes depends on the stimulus and the cell type.

On the basis of the results obtained in the present study, it is concluded that the ethanolic* O. paradoxa* extract, rich in flavonoids and phenolic compounds, may act predominantly as a potent inhibitor of* E. coli*- and PMA-induced phagocyte oxidative burst, the efficiency of which is cell-type- and stimulus-dependent, suggesting that attenuated ROS production by phagocytes might contribute to the anti-inflammatory properties of the extract polyphenols. Additionally, our studies shed novel insights into stimulatory properties of the extract in fMLP-activated monocyte and neutrophil oxidative burst. However, further studies on the establishment of the mechanisms behind dual stimulus-dependent effects of the extracts on activated phagocytes are required. This would be a valuable step in forming a more complete picture of the process by which the extract polyphenols affect activated monocytes and neutrophils.

## Figures and Tables

**Table 1:** The effect of ethanolic *O.  paradoxa * extract on respiratory burst in monocytes and neutrophils stimulated with *E*.*  coli*, PMA, or fMLP.

	Median (range)			*P* value		
	Control^#^ (no extract) *n* = 36	Group A blood + extract (0.1 mg/mL) *n* = 36	Group B blood + extract (0.25 mg/mL) *n* = 36	Control versus A	Control versus B	A versus B
**Monocyte**						
*E. coli *						
M_ROS_ [%]	26.05 (5.30–68.30)	5.05 (0.20–26.50)	2.10 (0.40–8.30)	<0.001∗∗∗	<0.001∗∗∗	<0.001∗∗∗
MFI	426 (304–693)	427.5 (250–669)	361 (233–644)	0.285	0.023∗	0.036∗
PMA						
M_ROS_ [%]	21.65 (2.80–85.00)	10.00 (0.40–47.10)	7.65 (0.20–14.70)	<0.001∗∗∗	<0.001∗∗∗	<0.01∗∗
MFI	538.00 (339–893)	568.75 (347–879)	484.50 (230–952)	0.712	0.068	0.107
fMLP						
M_ROS_ [%]	0.3 (0.1–2.1)	0.4 (0.1–2.7)	0.6 (0.1–8.2)	<0.01∗∗	<0.001∗∗∗	0.028∗
MFI	307 (241–515)	318 (234–664)	326 (233–415)	0.061	0.912	0.049∗
**Neutrophil**						
*E. coli *						
N_ROS_ [%]	87.55 (49.90–99.31)	61.50 (28.70–96.29)	52.10 (17.53–91.08)	<0.001∗∗∗	<0.001∗∗∗	<0.01∗∗
MFI	894 (504–1600)	619 (397–1169)	550 (377–1098)	<0.001∗∗∗	<0.001∗∗∗	<0.001∗∗∗
PMA						
N_ROS_ [%]	91.20 (46.8–99.70)	83.25 (55.8–99.30)	84.25 (47.7–98.60)	0.018∗	0.035∗	0.880
MFI	1178 (645–2995)	1232 (574–2594)	1233 (442–2699)	0.451	0.371	0.285
fMLP						
N_ROS_ [%]	3 (0.2–11.50)	3.25 (0.10–14.85)	3.95 (0.2–19.80)	0.276	0.014∗	0.017∗
MFI	328 (274–609)	330 (276–464)	340 (266–508)	0.118	0.545	0.314

^#^Control (blood + PMA or *E.  coli* or fMLP alone).

M_ROS_  [%] and N_ROS_  [%]: percentage of monocytes and neutrophils producing ROS, respectively; MFI: mean fluorescence intensity; fMLP: *N*-formyl-methionyl-leucyl-phenylalanine; PMA: phorbol-12-myristate-13-acetate.

*P < 0.05, **P < 0.01, and ***P < 0.001 as assessed by Wilcoxon's test.

**Table 2:** Antioxidant enzyme activities after treatment with the extract.

Enzyme	Control	*O. paradoxa* extract		*P*
		[0.1 mg/mL]	[0.25 mg/mL]	
CAT [U/mg of protein]	74.98 ± 17.93	75.27 ± 16.96	76.87 ± 18.76	ns
GPx [U/mg of protein]	0.16 ± 0.05	0.16 ± 0.25	0.17 ± 0.06	ns
SOD [% of inhibition]	32.40 ± 9.41	33.15 ± 8.10	35.80 ± 9.07	ns

Values represented as mean ± SD of three independent experiments.

CAT: catalase; GPx: glutathione peroxidase; SOD: superoxide dismutase.

ns: not significant as assessed by Wilcoxon's test.

**Table 3:** Characteristics of the extract polyphenolics identified by HPLC-MS analysis.

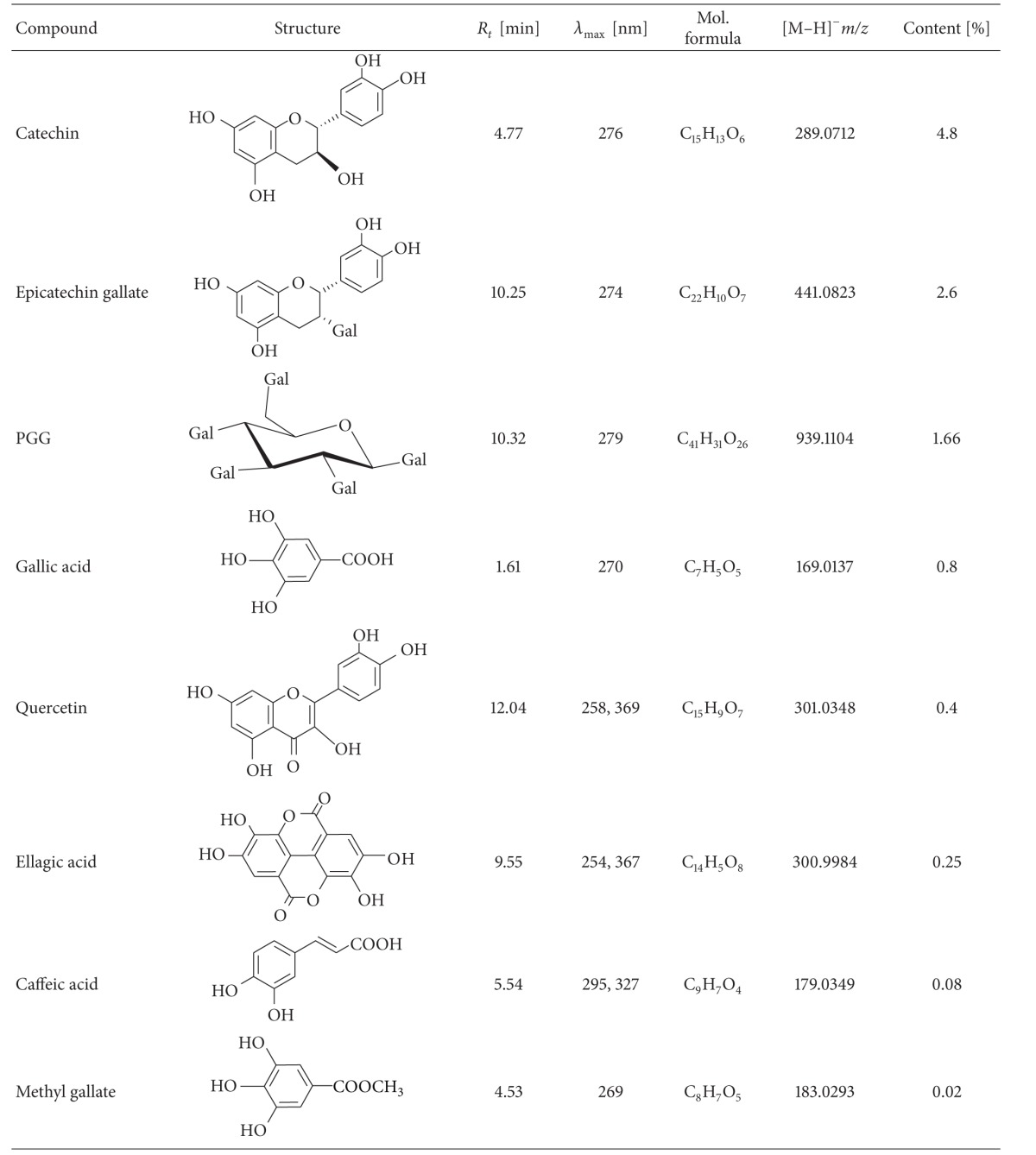

## Abbreviations

CAT: Catalase
fMLP: Formyl-methionyl-leucyl-phenylalanine
GPx: Glutathione peroxidase
HPLC: High-performance liquid chromatography
MFI: Mean fluorescent intensity
PGG: Penta-galloyl-*β*-(D)-glucose
PMA: Phorbol 12-myristate 13-acetate
ROS: Reactive oxygen species
SOD: Superoxide dismutase.
